# Integrin α11 cytoplasmic tail is required for FAK activation to initiate 3D cell invasion and ERK-mediated cell proliferation

**DOI:** 10.1038/s41598-019-51689-6

**Published:** 2019-10-25

**Authors:** Pugazendhi Erusappan, Jahedul Alam, Ning Lu, Cédric Zeltz, Donald Gullberg

**Affiliations:** 1 Department of Biomedicine and Center of Cancer Biomarkers, University of Bergen, Jonas Lies vei 91, N-5009 Bergen, Norway; 2 Institute for Experimental Medical Research, Oslo University Hospital and University of Oslo, Kirkeveien 166, 0450 Oslo, Norway; 3 Princess Margaret Cancer Center, University Health Network, 101 College Street, Toronto, ON M5G 1L7 Canada

**Keywords:** Extracellular matrix, Focal adhesion, Integrins, Integrin signalling

## Abstract

Integrin α11β1 is a collagen-binding integrin, which is receiving increasing attention in the context of wound healing and fibrosis. Although α11β1 integrin displays similar collagen specificity to α2β1 integrin, both integrins have distinct *in vivo* functions. In this context, the contribution of α11 subunit cytoplasmic tail interactions to diverse molecular signals and biological functions is largely unknown. In the current study, we have deleted the α11 cytoplasmic tail and studied the effect of this deletion on α11 integrin function. Compared to wild-type cells, C2C12 cells expressing tail-less α11 attached normally to collagen I, but formed fewer focal contacts. α11-tail-less cells furthermore displayed a reduced capacity to invade and reorganize a 3D collagen matrix and to proliferate. Analysis of cell signaling showed that FAK and ERK phosphorylation was reduced in cells expressing tail-less α11. Inhibition of ERK and FAK activation decreased α11-mediated cell proliferation, whereas α11-mediated cell invasion was FAK-dependent and occurred independently of ERK signaling. In summary, our data demonstrate that the integrin α11 cytoplasmic tail plays a central role in α11 integrin-specific functions, including FAK-dependent ERK activation to promote cell proliferation.

## Introduction

Integrins are heterodimeric cell surface receptors composed of non-covalently associated α and β subunits, which act as cell surface links to the extracellular matrix (ECM) and to other cells in dynamic cell-cell linkages^1^. Integrin subunits are composed of different domains with different functions. The extracellular domain of collagen-binding α integrin chains contain an inserted α-I domain, which is responsible for collagen binding without direct involvement of the β subunit. Whereas different integrin β chains display conserved regions including their cytoplasmic tail, the cytoplasmic tails of integrin α chains show little sequence similarity except for the very proximal membrane sequence GFFXR^2^. It is interesting in this context to note that α11 integrin lacks the conserved GFFXR sequence, but instead the α11-tail contains the GFFRS sequence^3^. A number of proteins have been found to bind to the conserved GFFXR sequence without displaying specificity for any particular α chain^2^. The conserved GFFXR site has been demonstrated to bind, for example, SHARPIN^4^, which keeps integrin in an inactive conformation. Other proteins reported to bind to the conserved membrane proximal region include Rab21, Nischarin and PP2A^2^. Deletion of GFFXR or mutation of Arginine in the GFFXR sequence render integrins constitutively active, suggested to occur as a result of breakage of a salt linkage between α chains and β chain^5^. If the deletion occurs beyond the GFFXR sequence, effects vary depending on the nature of the α chain and the cellular background^6^.

Molecular interactions of the integrin cytoplasmic tails can both regulate inside-out and outside-in signaling as well as strengthening the actin linkages^2,7^. The NPXY motifs located in the β subunits are important binding sites for talins and kindlins, both taking part in integrin inside-out signaling^8,9^. These important interactions in turn are regulated through binding of other proteins such as Dok1 and ICAP-1, to the same integrin β chain NPXY motifs. In addition, phosphorylation of the proximal NPXY motif appears to be a molecular switch to regulate tensin binding and localization of α5β1 to fibrillar adhesions^10^. More recent data have demonstrated that integrin α chains contribute to filamin A-, talin-, and kindlin-binding to the integrin β subunits^11–14^. Data are thus accumulating with indications that integrin α cytoplasmic tails take an active part in interactions of importance for integrin heterodimer function.

Careful analyses of mice lacking individual collagen-binding integrins show that the collagen-binding integrin receptors are dispensable for normal development, but suggest important roles for these receptors in tissue remodeling events occurring in wound healing, fibrosis and tumor-stroma interactions^15^. α11β1 integrin is a collagen receptor, which is the latest identified member of the integrin family^3,16,17^. Although α11 shows an overall sequence homology to other collagen-binding integrin α chains of the β1 subfamily and also displays similar collagen specificity as α2β1 integrin, α11β1 integrin *in vivo* has functions distinct from the other collagen-binding integrins^18–22^. This suggests that α11 cytoplasmic tail may regulate α11 functions. The role of cytoplasmic tails of collagen-binding integrins has been studied extensively in the 1990s by the group of Hemler *et al*. Deletion of the α2 integrin cytoplasmic tail in K562 and RD cells (both requiring integrin activation) demonstrated that the α2 -tail-less integrin showed reduced adhesive activity on collagen I, in a manner suggesting an activation defect^23^. In chimeric experiments where again the α2 cytoplasmic tail was replaced with the tail of other integrins, it was demonstrated that chimeric α2 integrins with α5-tail (Xα2 Cα5) could mediate collagen gel contraction, whereas chimeric Xα2 Cα4 failed to mediate contraction, but instead promoted cell migration^24^. Already at this time it was speculated that “α subunit cytoplasmic domains, probably acting in concert with their associated β subunit, also have important but distinct roles and perhaps eventually will be shown to interact with distinct set of intracellular proteins”^24^. More recent data, using more sensitive assays, analyzing chimeras of all 12 α chains of the β1 integrin subfamily confirm that cytoplasmic tails of integrin α-chains do affect integrin inside-out activation, but that this varies greatly between different integrin α chains^25^. Deletion experiments are thus not easy to interpret since one also has to consider possible modulatory effects on integrin α/β chain interactions.

In the current study, we have deleted the α11 cytoplasmic tail and studied the effect of this deletion on α11 integrin function. Our data show that the integrin α11 cytoplasmic tail is dispensable for cell attachment but is essential for focal adhesion formation, ERK-dependent cell proliferation, cell migration and reorganization of 3D collagen matrices.

## Results

### Generation and expression of a human integrin α11-tail-less variant

**Figure 1:**
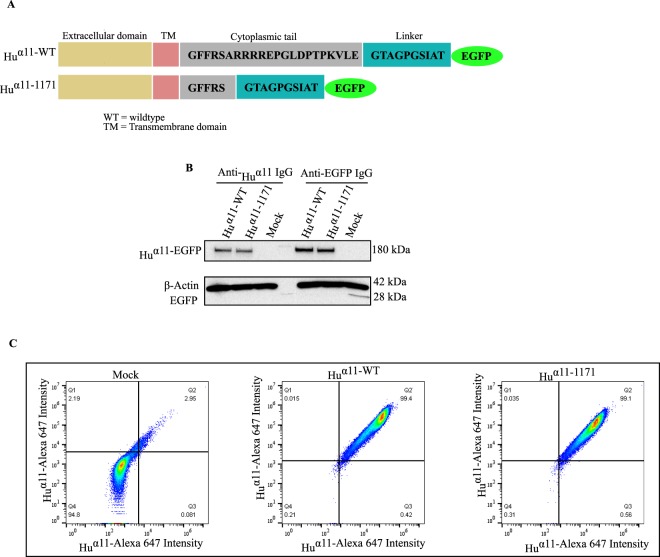
Generation and expression of integrin α11 variants in C2C12 cells. (**A**) Schematic illustration showing the amino acid sequences in the linker and the cytoplasmic tail of _Hu_α11 variants. (**B**) Western blot showing total protein expression of _Hu_α11-EGFP in C2C12 cells transfected with _Hu_α11-WT-EGFP and _Hu_α11-1171-EGFP (full size immunoblot is shown in supplementary) (**C**). FACS analysis of the cell surface expression α11 in _Hu_α11-WT, _Hu_α11-1171 and mock transfected cells (Mock).

In order to identify the role of the α11 cytoplasmic tail, a mutant variant (_Hu_α11-1171) with a deletion of the terminal 17 amino acids in the cytoplasmic tail of human integrin α11 (_Hu_α11) was generated. Since the antibodies used to detect human α11 react with the cytoplasmic tail^26^, we have EGFP-tagged the integrin carboxy terminus using a 10 amino acid linker to avoid interference of the EGFP tag. Horwitz *et al*. pioneered this strategy for integrin α5 and the resultant tagged α5 integrin was characterized in detail without any evidence of artifacts due to the EGFP tag^27^. Full-length (_Hu_α11-WT) and tail-less (_Hu_α11-1171) _Hu_α11 variants were tagged with enhanced green fluorescence protein (EGFP) and expressed in C2C12 mouse myoblasts, which do not express any collagen-binding integrins^28^ (Fig. 1A). Based on EGFP intensity, the transfected cells were sorted by flow cytometry with uniform gating to obtain similar expression levels of EGFP. The expression of comparable levels of _Hu_α11-EGFP in the total protein lysates was confirmed by immunoblotting, either with an anti-_Hu_α11 polyclonal antibody or with an anti-EGFP antibody (Fig. 1B). Comparable expression levels of _Hu_α11-EGFP at the cell surface were also confirmed, using mock transfected cells (Mock, empty GFP vector) as a negative control (Fig. 1C).

### Integrin α11 cytoplasmic tail is dispensable for cell adhesion but mediates focal adhesion formation, collagen reorganization, cell migration and cell proliferation

**Figure 2:**
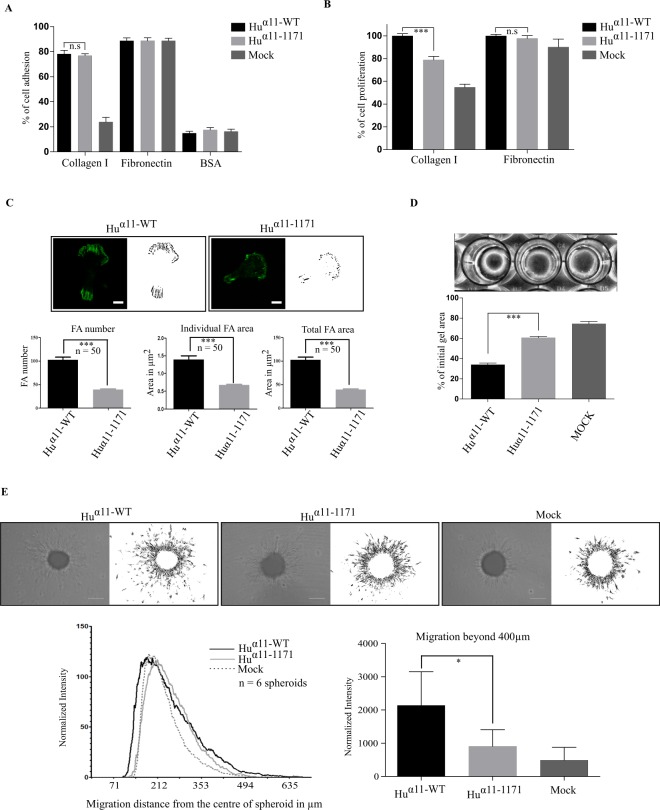
Integrin α11 cytoplasmic tail is not involved in cell adhesion but mediates focal adhesion formation, collagen reorganization, cell migration and cell proliferation. (**A**) Role of α11-tail in cell adhesion. _Hu_α11-WT, _Hu_α11-1171 and mock transfected cells (Mock) were allowed to attach on collagen I or fibronectin or BSA in serum-free conditions for 50 mins. Attached cells were fixed, stained with 0.1% crystal violet and absorbance was read at 595 nm. (**B**) Role of α11-tail in cell proliferation. _Hu_α11-WT, _Hu_α11-1171 and Mock cells were allowed to attach on collagen I or fibronectin in reduced serum condition for 24 hours. Attached cells were fixed, stained with 0.1% crystal violet and absorbance was read at 595 nm. (**C**) Role of α11 tail in focal adhesion formation. Cells were allowed to attach collagen I for 120 mins. Cells were fixed with 4% PFA and focal adhesions were imaged using TIRF microscopy and quantified. Scale bar: 10 µm. (**D**) Role of α11-tail in collagen reorganization. _Hu_α11-WT, _Hu_α11-1171 and Mock cells were mixed with collagen I solution and allowed to contract for 16 hours. Gel diameters were measured, and percentage of initial gel area was calculated. (**E**) Role of α11-tail in spheroid migration. Homospheroids of _Hu_α11-WT, _Hu_α11-1171 and Mock cells were embedded in collagen I gel and spheroid migration was quantified after 24 hours. Radial profile plot depicts the radial cell intensity from the center of the spheroid and the intensity of cells that have migrated beyond 400 µm was calculated. Scale bar: 200 µm. Data shown are pooled from triplicates of at least three independent experiments for cell attachment, cell proliferation and collagen gel contraction. Results were expressed as mean ± standard deviation of at least three replicates from one representative experiment of at least three independent experiments. Statistical significance was assessed by two tailed, unpaired t-tests and P-values are expressed as ***P < 0.001; **P < 0.01 and *P < 0.05.

To examine the role of integrin α11 cytoplasmic tail in cell adhesion, _Hu_α11-WT and _Hu_α11-1171 cells were allowed to attach to collagen I or fibronectin using BSA coated wells as negative control. Mock transfected cells did not adhere to collagen, whereas _Hu_α11-WT and _Hu_α11-1171 cells adhered equally well to collagen I and fibronectin. This suggests that the deletion of 17 amino acids in the cytoplasmic tail of _Hu_α11 had no apparent negative influence on integrin activation or cell adhesion to collagen I (Fig. 2A). However, _Hu_α11-1171 cells displayed fewer focal adhesions after 2 hours, with a reduction of 50% in the total area of focal adhesions (Fig. 2C) and 35% reduction in cell spreading (Supplementary Fig. 2). This indicates that cytoplasmic tail of integrin α11 is involved in mediating cell adhesion signaling. To confirm this hypothesis, we assessed the ability of _Hu_α11-1171 cells in mediating: collagen gel reorganization, cell migration and proliferation.

When cells were allowed to attach on collagen I for 24 hours in low serum conditions, _Hu_α11-1171 cells displayed a significant reduction in cell proliferation compared to _Hu_α11-WT cells, but not on fibronectin, suggesting that the α11 cytoplasmic tail-mediated signaling is involved in the regulation of cell proliferation (Fig. 2B). We further examined the ability of these cells to contract 3D collagen I lattices, a process previously shown to be α2β1- and α11β1- mediated^18,28,29^. Sixteen hours after the contraction was initiated, the _Hu_α11-1171 cells displayed 50% reduction in collagen contraction compared to _Hu_α11-WT cells (Fig. 2D).

We also investigated the role of the α11 cytoplasmic tail in cell migration using a spheroid migration model in a 3D collagen matrix. The radial cell density profile of the spheroid was analyzed from the center of the spheroid to quantify migrated cells in relation to their distance of migration. _Hu_α11-WT cells migrated out 50% more than _Hu_α11-1171 and mock transfected cells, beyond 400 µm from the center of the spheroid (Fig. 2E). Interestingly, the size of the spheroid core for the _Hu_α11-WT cells was smaller than that observed for spheroids formed from the _Hu_α11-1171 and Mock cells. These results indicate that the α11 cytoplasmic tail is indeed essential to mediate cell proliferation, collagen reorganization and cell migration.

### Integrin α11 cytoplasmic tail contributes to FAK and ERK activation

**Figure 3:**
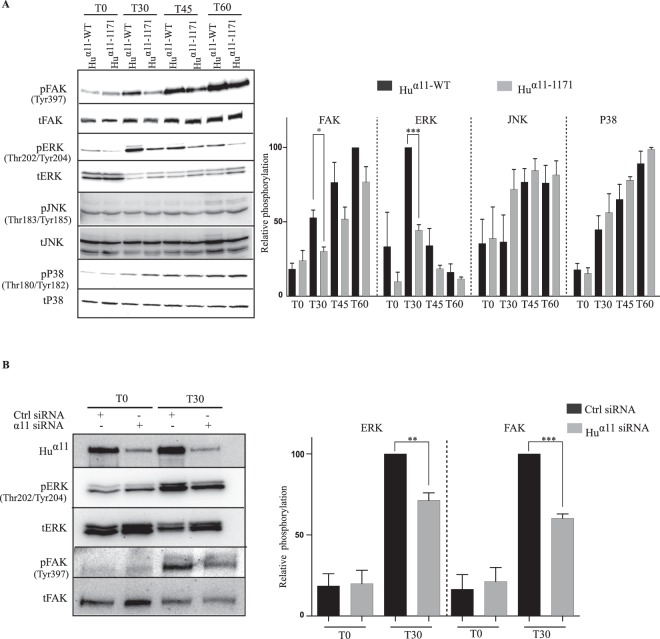
Integrin α11 cytoplasmic tail contributes to FAK and ERK activation. (**A**) Serum-starved _Hu_α11-WT and _Hu_α11-1171 cells were plated on collagen I in serum-free conditions and cells were lysed at different time points (T0, T30, T45 and T60). Total and phosphorylated levels of FAK^Y397^, ERK, p38, JNK were detected by Western blotting and the protein bands were quantified by densitometry analysis (full size immunoblots are shown in supplementary). (**B**) Human gingival fibroblasts (hGFs) were transfected with control (ctrl) siRNA or α11 siRNA (SMARTpool) and 48 hours post transfection, cells were serum-starved and plated on collagen I in serum-free conditions. After 30 mins, cells were lysed, and the lysates were analyzed by western blotting. Protein bands were quantified by densitometry analysis. Statistical significance was assessed by two tailed, unpaired t-tests and P-values are expressed as ***P < 0.001; **P < 0.01 and *P < 0.05.

Localization of focal adhesion kinase (FAK) in focal adhesions and its autophosphorylation at Tyrosine residue 397 (FAK^Y397^) is a primary event in integrin signaling leading to cell migration and proliferation^30–33^. Analysis of FAK^Y397^ activation at different time points revealed that _Hu_α11-1171 cells demonstrated less FAK^Y397^ activation than _Hu_α11-WT cells at 30 and 45 minutes on collagen I (Fig. 3A). Since other collagen-binding integrins have previously been shown to activate MAPK pathways, we investigated activation of ERK, p38 and JNK following attachment of cells to collagen I^34–36^. Interestingly, _Hu_α11-1171 cells showed a strong reduction in ERK activation compared to _Hu_α11-WT cells on collagen I (Fig. 3A). In contrast, activation of p38 and JNK was not affected by the deletion of the α11 cytoplasmic tail. To confirm the role of integrin α11 in FAK and ERK activation in primary cells, human gingival fibroblasts (hGF) were transfected with control siRNA (Ctrl) or α11 siRNAs (Fig. 3B and supplementary Fig. 3A). The knockdown of α11 did not affect expression of the collagen-binding integrins α1 and α2 chains (Supplementary Fig. 3B,C). Phosphorylated FAK^Y397^ and ERK levels were only reduced in α11 siRNA-treated hGF (Fig. 3B and supplementary Fig. 3A). These results demonstrate that cytoplasmic tail of integrin α11 contributes to FAK and ERK activation.

### FAK^Y397^ activation is involved in integrin α11 cytoplasmic tail-mediated cell proliferation and migration

**Figure 4:**
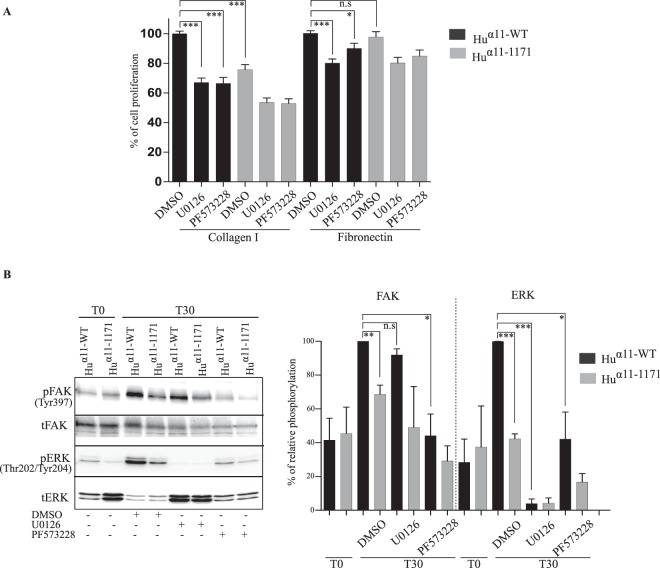
FAK^Y397^ activation is involved in integrin α11 cytoplasmic tail-mediated cell proliferation and migration. (**A**) Effect of FAK and ERK inhibition in α11-mediated cell proliferation. _Hu_α11-WT and _Hu_α11-1171 cells were allowed to attach on collagen I or fibronectin in presence of either DMSO or U0126 (20 µM) or PF573228 (10 µM) in reduced serum conditions for 24 hours. Attached cells were fixed, stained with 0.1% crystal violet and absorbance was read at 595 nm. Results were expressed as mean ± standard deviation of at least three replicates pooled from three independent experiments. (**B**) Integrin α11 tail-mediated ERK activation is dependent on FAK^Y397^ activation. Serum starved _Hu_α11-WT and _Hu_α11-1171 cells were treated with DMSO or U0126 or PF573228 and allowed to attach on collagen I for 30 minutes. After 30 minutes, cells were lysed, and the lysates were analyzed for total and phosphorylated levels of FAK^Y397^ and ERK by Western blotting. Protein bands were quantified by densitometry analysis and data shown are pooled from at least three independent experiments (Full size immunoblots are shown in supplementary). Statistical significance was assessed by two tailed, unpaired t-tests and P-values are expressed as ***P < 0.001; **P < 0.01 and *P < 0.05.

We next examined if the reduced cell functions observed with the α11-tail-less cells was due to the defect in FAK and ERK activation. Inhibition of FAK^Y397^ activation by PF573228 inhibited cell proliferation in both _Hu_α11-WT and _Hu_α11-1171 cells, on collagen I. Similarly, inhibition of ERK activation by U0126 also inhibited α11β1-mediated cell proliferation on collagen I, implying that both FAK^Y397^ and ERK activation are required for α11β1-mediated cell proliferation (Fig. 4A). Inhibition of both FAK and ERK activation weakly decreased cell proliferation in _Hu_α11-WT cells on fibronectin, suggesting that cell proliferation mediated by fibronectin-binding integrins is less dependent on these signaling molecules than α11β1-mediated cell proliferation. In order to understand the overlapping function of FAK and ERK in α11β1-mediated cell proliferation, we investigated the relationship between FAK and ERK activation. Inhibition of FAK^Y397^ activation by PF573228 significantly inhibited ERK activation 30 minutes after cells attached to collagen (Fig. 4B). In contrast, inhibition of ERK activation by U0126 did not affect FAK^Y397^ activation, indicating that FAK^Y397^ phosphorylation is required for ERK activation.

**Figure 5:**
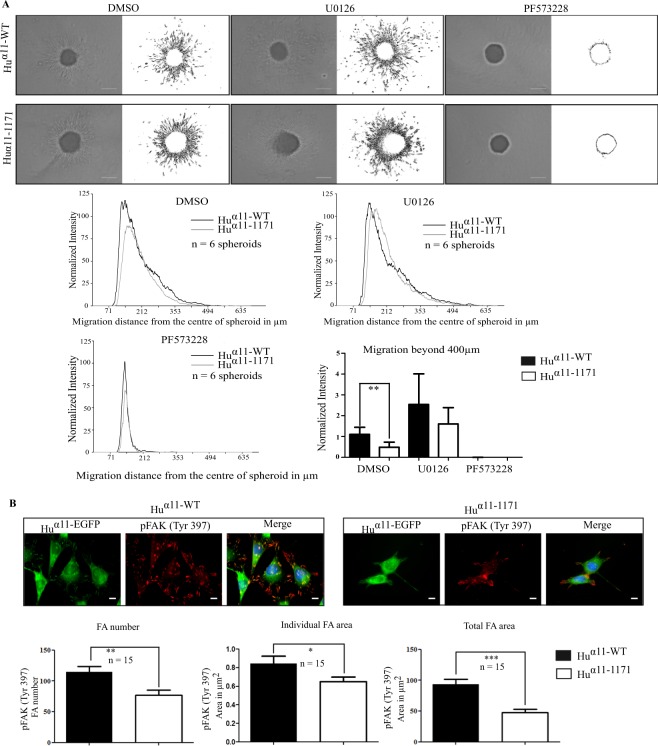
Integrin α11 cytoplasmic tail is involved in focal adhesion formation and FAK^Y397^ activation. (**A**) Effect of FAK and ERK inhibition in α11-mediated spheroid migration. Homospheroids of _Hu_α11-WT and _Hu_α11-1171 cells were embedded into collagen I gel and treated with either DMSO or U0126 or PF573228. Spheroid migration was quantified after 24 hours. Radial profile plot depicts the radial cell intensity from the center of the spheroid and the intensity of cells that have migrated beyond 400 µm was calculated. Scale bar: 200 µm. (**B**) Localization of phospho-FAK^Y397^ in focal adhesions. _Hu_α11-WT and _Hu_α11-1171 cells were allowed to attach on collagen I for 120 minutes. Cells were fixed with 4% PFA and stained for phospho-FAK^Y397^. Focal adhesions positive for phospho-FAK^Y397^ were quantified using ImageJ. Scale bar: 10 µm. Results were expressed as mean ± standard deviation of at least three replicates from one representative experiment of at least three independent experiments. Statistical significance was assessed by two tailed, unpaired t-tests and P-values are expressed as ***P < 0.001; **P < 0.01 and *P < 0.05.

In spheroid migration assays, inhibition of FAK^Y397^ phosphorylation, completely abrogated migration for both _Hu_α11-WT and _Hu_α11-1171 cells (Fig. 5A). Contrariwise, inhibition of ERK in spheroid assay did not inhibit migration beyond 400 µm of both _Hu_α11-WT and _Hu_α11-1171 cells, suggesting that in contrast to cell proliferation, ERK activation is not involved in integrin α11-mediated cell migration in this 3D model. Furthermore, neither inhibition of FAK^Y397^ activation nor ERK activation inhibited serum-stimulated collagen gel contraction (data not shown).

Since FAK is localized in focal adhesions, we investigated the phosphorylation of FAK^Y397^ in focal adhesions. Similar to our previous results with immunoblotting, we observed significantly reduced amounts of phosphorylated FAK^Y397^ positive adhesions for _Hu_α11-1171 cells as compared to _Hu_α11-WT cells (Fig. 5B). These findings suggest that the reduced activation of FAK ^Y397^ in _Hu_α11-1171 cells is due to fewer focal adhesions in these cells per se.

To summarize, our results strongly suggest that the cytoplasmic tail of integrin α11 is essential to stabilize focal adhesions that in turn mediate FAK^Y397^ activation involved in cell proliferation and cell migration.

## Discussion

Although integrin cytoplasmic tails are relatively short, they are able to exert control of integrin activity and mediate a number of interactions of importance for integrin function^7,8^. There are in total more than 20 integrins and various research groups have independently deleted cytoplasmic tails and expressed the mutated variants with different results^6,37–39^. When integrin β-tails are expressed as chimeric proteins with non-integrin transmembrane- and extracellular parts, the β1 integrin tail sequence can direct chimeric proteins into focal adhesions^40^. When integrin α-tails are deleted, some α-tail-less heterodimers localize to focal adhesions in a ligand-independent manner^41–43^ and often become constitutively activated.

In the current study, we have used EGFP-tagged α11 integrins, in a strategy where the tag is separated by a 10 amino acid linker. Like for EGFP tagged α4 and α5 integrins we find no evidence that addition of the tag interferes with integrin function^27,44^. It could be argued that addition of a tag on tail-less integrin would be more likely to interfere with integrin function than a tag added to full-length integrin. However, independent experiments suggest that the loss of α11 integrin activity seen in the tail-less variant can be duplicated by a specific point mutation in the α11-tail, excluding that non-specific interference of the EGFP-tag (data not shown).

In our present study, we found that deletion of α11 cytoplasmic tail led to reduced focal adhesion formation, reduced cell spreading, reduced collagen gel contraction, reduced cell migration in a 3D context and reduced ERK-dependent cell proliferation, but that the α11-tail was dispensable for cell adhesion under the conditions used. Deletion of α1 cytoplasmic tail in fibroblastic 3T3 cells leaves cell adhesion to collagen IV unaffected but results in ligand-independent focal contact localization^45^, whereas in endothelial cells α1-tail deletion leads to reduced adhesion to collagen IV^36^. Deletion of α2 cytoplasmic tail reduces cell adhesion to collagen I in RD rhabdomyosarcoma cells, but this effect can be overcome by ions or ligand concentration, suggesting that a major function of the cytoplasmic α2-tail is to mediate intracellular inside-out activation events, and that α2 deletion effects on cell attachment can be overcome by activating the extracellular domain^23^. The reduced number of focal contacts seen in α11-tail-less expressing cells probably reflects a contribution of α11-tail to a cytoskeletal linkage, whose nature remains to be established, rather than need for α11-tail in integrin activation. *In vivo* integrin α2β1 is expressed in platelets and hematopoietic cells^46^ where integrin activation is essential, whereas α11β1 is mainly expressed on fibroblastic cells^26^ where β1 integrins are constitutively activated^47^.

Here we showed that interaction of α11β1 with collagen I mediated ERK signaling. This signaling is thus similar to that observed for α1 (although the preferred ligand for α1β1 is collagen IV^48^), but is different than for α2β1-mediated signaling, which occurs mainly via p38 in 3D collagen I matrix^34^. Interestingly, in mouse endothelial cells, limited α2-dependent p38 signaling is observed^36^. These data suggest for collagen-binding integrins that the presence of cell-dependent factors influence which MAPK signaling pathway will be activated upon collagen ligation. siRNA knockdown of α11 reduced FAK and ERK activation, supporting that α11-mediated ERK signaling is central in fibroblasts, which is the major cell type expressing α11. Previous studies have demonstrated α11-dependent ERK and PI3K phosphorylation in mesenchymal stem cells expressing multiple collagen-binding integrins^49^. However, in our cell system (C2C12 cells lacking other collagen receptors than the overexpressed α11β1), we failed to detect α11-dependent PI3K activation (data not shown).

Blocking α11-dependent cellular signaling in C2C12 and human gingival fibroblasts cells also blocked ERK-dependent cell proliferation. A majority of the α11-dependent ERK signaling appeared to be dependent on FAK, since FAK inhibition also attenuated the α11-dependent ERK signaling. In the case of α1, FAK independent ERK signaling via Shc has been noted^50^. Later studies have demonstrated that FAK may enhance and prolong integrin-mediated activation of ERK through p130 (CAS), Crk, and Rap1 in cells expressing B-Raf^51^. α2-mediated p38 activation has been suggested to depend on specific residues within the α2 integrin subunit cytoplasmic domain^52^, and independent experiments failed to record FAK activation in 3D collagen gel under conditions of α2-mediated p38 activation^34^.

To analyze cell migration in 3D collagen gel we used a spheroid assay. Cell migration^53^, MMP-induction^54^ and collagen gel remodeling^55^ has been shown to depend on ERK signaling in some conditions. In our study, ERK inhibition did not impair cell migration in a collagen matrix. ERK inhibition could attenuate G-protein dependent integrin inhibition as has been reported for α2β1 integrin-dependent cell migration in smooth muscle cells^56^.

Finally, the collagen gel contraction was not affected by ERK or FAK inhibition suggesting that an alternative signaling pathway is operative in the C2C12 cells overexpressing α11. We have previously demonstrated that TGF-β-dependent contraction of floating collagen lattices by dermal fibroblasts depends on α11- and JNK- signaling^19^. This signaling pathway might be restricted to dermal fibroblasts or depend on relative levels of crucial components in non-canonical TGF-β signaling pathway being present in the cells. Previous studies have demonstrated that thrombospondin 1 in scleroderma fibroblasts can activate TGF-β to stimulate ERK-dependent collagen contraction^57^. Since αvβ3 signals via ERK, it is possible that αvβ3 mediates this collagen gel contraction under these conditions^58^. ERK activation has been shown to stimulate phosphorylation of MLC and in this way contribute to collagen lattice contraction^53^, but in our experiments pharmacological inhibition of ERK in α11-C2C12 cells failed to inhibit contraction.

In summary, our data suggest that the unique functions of α11 that separates it from other collagen-binding integrins is in part due to its cytoplasmic tail, which is needed for efficient focal contact formation, cell spreading, cell proliferation, cell migration and collagen remodeling.

## Materials and Methods

### Cell culture

Mouse C2C12 mouse satellite cells were provided by Prof. Anna Starzinski-Powitz (Goethe-Universität, Frankfurt am Main, Germany) and Phoenix 293 cells were provided by Prof. James Lorens, University of Bergen. Primary human gingival fibroblasts (hGF) were isolated from healthy gingival tissues as described earlier^59^. MRC5 human lung fibroblasts (American Type Culture Collection) were obtained from Robert Lafyatis laboratory (University of Pittsburgh Medical Center, Pittsburgh, PA, USA). Cells were cultured at 37 °C in Dulbecco’s modified Eagle’s medium (DMEM; Gibco®, Invitrogen) with 10% fetal bovine serum (FBS; Gibco®, Invitrogen), 1% penicillin-streptomycin (PEST; Sigma-Aldrich) and 5 µg/ml plasmocin (InvivoGen). Human gingival fibroblasts were grown from biopsies obtained during oral surgery after obtaining informed consent and in accordance with guidelines and regulations at the Department of Prosthetic Dentistry, Karolinska Institute, Stockholm in the 1990s following approval of experimental protocols by local ethics committee at faculty of Odontology, Karolinska institute and were kindly provided by Prof. Kamal Mustafa (University of Bergen)^60^.

### Generation and expression of integrin α11 variants in C2C12 mouse satellite cells

To construct pBABE *ITGA11* retroviral expression constructs, pBABE-puro-Itga11 plasmid, pBJ1- _Hu_α11-WT-EGFP and pBJ1- _Hu_α11-1171-EGFP (for detail, see Supplementary information) were used as templates. The ITGA11-EGFP cDNAs from pBJ1-_Hu_α11-WT-EGFP and pBJ1-_Hu_α11-1171-EGFP were excised with *XhoI* (blunted) and *EcoRI* and subcloned into pBABE-puro-Itga11 plasmid at *BamHI* (blunted) and *EcoRI* sites. The constructs were transfected into Phoenix 293 packaging cell line with X-tremeGENE 9 transfection reagent (Roche Diagnostics GmbH), according to manufacturer’s instructions. The viral supernatant medium was collected after 48 hours post transfection. C2C12 cells, cultured on 6-well plates were infected with viral supernatants containing polybrene at 5 µg/ml by spinfection at 1200 g for 90 minutes. After 36 hours, the culture medium was changed to a selection medium containing 2 µg/ml of puromycin. In addition, the cell populations with similar levels of EGFP intensity were sorted by Fluorescence-activated cell sorting (FACS).

### Estimation of cell surface protein expression by FACS

FACS was performed at The Molecular Imaging Centre (MIC), University of Bergen. Cells were detached with Trypsin-EDTA (0.05% Trypsin and 0.02% EDTA; Gibco®, Invitrogen) and neutralized with DMEM containing 10% FBS. The cell suspension was filtered with a 40 µm syringe filter and 2 × 10^6^ cells were used for the analysis. Cells were washed twice with PBS for 5 minutes at 210 × g with phosphate buffered saline (PBS) and the cell pellet was fixed with 2% paraformaldehyde/PBS for 10 minutes. After fixation, cells were washed three times with PBS and blocked with 2% Bovine serum albumin (BSA/PBS) for 30 minutes in room temperature (RT). Cells were incubated with mouse anti- _Hu_α11 IgG (mAb 203E3)^61^ at a final concentration of 5 µg/ml in 2% BSA/PBS for 60 minutes at 37 °C. After washing three times with PBS, cells were incubated with goat anti-mouse IgG conjugated with Alexa fluor® 647 for 60 minutes in RT. Cells were washed 3X with PBS and analyzed using FACS Accuri for the intensity of Alexa fluor® 647 by using uniform gating for all samples and data was analyzed using FLOWJO computer software for FACS analysis (FLOWJO, LLC).

### Cell adhesion assay

Forty-eight-well plates were coated with fibronectin (1 μg/cm^2^: Sigma-Aldrich) or collagen type I (5 μg/cm^2^: Bovine PureCol®, Advanced BioMatrix) and incubated for 2 hours at 37 °C. After washing with PBS, the plates were blocked with 2% BSA for 1 hour at 37 °C. Cells were washed three times with DMEM and 1 × 10^5^ cells/well were cultured for 50 minutes at 37 °C. Unattached cells were removed carefully by washing three times with PBS containing Ca^2+^ and Mg^2+^. Cells were then fixed with 96% ethanol for 10 minutes at room temperature followed by staining with 0.1% crystal violet for 20 minutes at room temperature. Plates were washed three times with distilled water and the cells were lysed with 1% Triton X-100 for 5 minutes. The lysates were transferred to a 96-well plate and absorbance was read at 595 nm.

### Cell proliferation assay

Cells were seeded on 24-well plates coated with fibronectin (1 μg/cm^2^) or collagen type I (5 μg/cm^2^) and cultured for 24 hours in DMEM containing 1% FBS. Cells were washed with PBS and fixed with 96% ethanol for 10 minutes at room temperature followed by staining with 0.1% crystal violet for 20 minutes at room temperature. After washing three times with distilled water, the cells were lysed with 1% Triton X-100 for 5 minutes and lysates were transferred to a 96-well plate. The absorbance was read at 595 nm. For inhibition experiments, cells were incubated with U0126 (20 µM; Sigma-Aldrich) or PF573228 (10 µM; Sigma-Aldrich). Data were normalized considering proliferation of wild-type cells as 100% on collagen and fibronectin.

### Collagen gel contraction

Collagen gel contraction was performed as described earlier^28,59^. In brief, 24-well plates were blocked with 2% BSA, overnight at 37 °C and then washed three times with PBS. Collagen solution was prepared by mixing 5 parts of DMEM 2 × (SLM-202-B, Merck Millipore), one part of 0.2 M Hepes at pH.8.0 and 4 parts of Collagen I (3 mg/ml; Bovine PureCol®, Advanced BioMatrix). Collagen solution was then mixed with cells to obtain a final concentration of 1 × 10^5^ cells/ml. To each well, 400 µl of cell-collagen suspension was added and allowed to polymerize for 90 minutes at 37 °C. Polymerized collagen gels were floated with 400 µl of DMEM containing 0.5% FBS. Gel diameters were measured using a ruler and the percentage of the initial gel area was calculated.

### Immunocytofluorescence

Glass bottom dishes (3.5 mm, MatTek) were coated with collagen type I (50 μg/cm^2^) for 60 minutes at 37 °C. Dishes were washed three times with PBS and 2 × 10^5^ cells were cultured for 2 hours in DMEM with 10% FBS. Cells were then fixed with 4% paraformaldehyde/PBS for 10 minutes at RT and followed by washing three times with PBS for 5 minutes per wash. Focal adhesion images were captured using Nikon Total Internal Reflection Microscope (TIRFM).

For phospho FAK^Y397^ staining experiments, coverslips (12 mm, 1.5 H; Marienfeld) were coated in 24-well plates with collagen type I (50 μg/cm^2^) for 60 minutes at 37 °C. Cover slips were washed three times and blocked with 2% BSA/PBS for 1 hour at 37 °C. Later, 4 × 10^4^ cells were seeded per well in serum-free conditions. Cells were cultured for 2 hours and fixed with 4% paraformaldehyde/PBS for 10 minutes at RT. Cells were permeabilized in 0.5% Triton X-100 /PBS buffer for 5 minutes and blocked with 5% BSA/PBS containing 0.1% Triton X-100 for 1 hour at RT. Next, cover slips were incubated with polyclonal rabbit anti-phospho FAK^Y397^ IgG (1:400; 44-624, Biosource) in 5% BSA/PBS with 0.1% Triton X-100 for 1 hour at 37 °C. After washing with 0.05% Tween-20/PBS, cover slips were incubated with Alexa fluor® 594 conjugated goat anti-rabbit IgG (1:400, Jackson ImmunoResearch) for 1 hour at RT. Later, coverslips were incubated with DAPI (0.25 μg/ml, Invitrogen) and mounted with ProLong Diamond Antifade mounting medium (Thermo Scientific). Cells were visualized under a Zeiss Axioscope fluorescence microscope and pictures were acquired with a digital AxioCam MRm camera.

### Spheroid preparation and migration assay in 3D collagen gel

Homospheroids were made with C2C12 cells using hanging drop method as described earlier^62^. In short, C2C12 cells were harvested and suspended in culture medium to have a final concentration of 1 × 10^6^ cells/ml. Approximately, 28 drops of cell suspension (25 µl/drop; 2.5 × 10^4^ cells) were made on the lid of a 10 cm Petri dish, containing cell culture medium. The lid was carefully inverted over the Petri dish bottom, without disturbing the drop form. The spheroids were cultured for 3 days under regular cell culture conditions. Collagen I solution was prepared as described in collagen gel contraction assay and 100 µl of collagen I solution was added onto a 96-well plate and incubated for 15 minutes at 37 °C. One spheroid was embedded per well and the spheroid-collagen gel was allowed to polymerize for 90 minutes at 37 °C. After polymerization, 100 µl DMEM was added to each well and cultured for 24 hours. Spheroids were visualized under an inverted light microscope (Leica DMIL) and images were captured. When indicated, DMSO or 20 µM U0126 or 10 µM PF573228 were added to the medium.

### SDS-PAGE and western blotting

Cells were seeded on 6-well plates and cultured until confluency. Later, cells were lysed with buffer containing 0.5% Nonidet P-40, 20 mM Tris-HCl pH7.4, 150 mM NaCl, 1 mM MgCl_2_, 1 mM CaCl_2_ and proteinase inhibitor complete (Roche, Germany). Protein concentration was determined by BCA assay. Protein samples of 20 μg from each clone, mixed with SDS sample buffer (Biorad) containing 2% of 2-β-mercaptoethanol were loaded and separated by 6% SDS-polyacrylamide gel. Then, the purified proteins were transferred to a PVDF membrane using iBlot® system (Invitrogen). Membranes were analyzed with mouse anti-EGFP IgG (1:2000, Clonetech) or polyclonal rabbit anti- _Hu_α11 IgG (PA5-23897, Thermo Fischer Scientific) or mouse anti-β-actin IgG (1:5000, Sigma-Aldrich). Blots were developed using ECL system (Pierce protein research products) and ChemiDoc XRS (Bio-Rad).

For signaling experiments, cells were cultured overnight with reduced serum conditions (1% FBS) and serum starved for 3 hours before the experiments. Trypsinized cells were neutralized with DMEM containing Soyabean trypsin inhibitor (50 µg/ml). Cells were washed three times with DMEM and incubated for 45 minutes on rotator at RT. 10^6^ cells were seeded on to 6-well plates pre-coated with collagen (5 μg/cm^2^) and blocked with 2% BSA/PBS, as described previously. For T0 samples, cells were lysed directly with 1X SDS sample buffer. For the remaining samples, cells in each were lysed in 1X SDS sample buffer after 30 min (T30), 45 min (T45) and 60 min (T60). Protein lysates were separated by SDS-PAGE and analyzed by western blotting using the following antibodies: Rabbit monoclonal anti-phospho p44/42 MAPK IgG (20G11), rabbit monoclonal anti-p44/42 MAPK IgG (137F5), rabbit anti-FAK IgG (#3285), rabbit monoclonal anti-phospho-SAPK/JNK IgG (81E11), rabbit anti-SAPK/JNK IgG (#9252), mouse monoclonal anti-phospho p38 IgG (28B10), rabbit anti-p38 IgG (#9212) from Cell Signaling Technology and rabbit anti-phospho FAKY397 IgG from Biosource. Relative protein expression was quantified using Image Lab™ Software (Bio-Rad).

### Integrin α11 silencing with siRNA

Primary hGFs were harvested and 5 × 10^5^ cells were plated on 10 cm culture dishes 30 min prior to transfection. Cells were transfected with SMARTpool ON-TARGET plus ITGA11 siRNA (L-008000-00-0005, Dharmacon) or Individual ON-TARGET plus ITGA11 (J-008000-10, Dharmacon) or ON-TARGET plus Non-Targeting siRNA (D-001810-02-05, Dharmacon) at a final concentration of 20 nM with HiPerfect transfection reagent. After 48 hours, cells were serum starved for overnight. Cells were prepared as described for signaling experiments. Six-well plates were coated with thin film fibrillar collagen I gel prepared using the collagen I solution described in collagen gel contraction section and allowed to polymerize for 60 min at 37 °C. Cells were harvested and 5 × 10^5^ cells were plated on to each 6-well and lysed as described above for SDS-PAGE and Western blotting analysis. Western blots stained with anti-phospho p44/42 MAPK IgG were reprobed with a custom-made mouse monoclonal anti- _Hu_α11 IgG, mAb 210F4 (Supplementary Fig. 1) to confirm the silencing of ITGA11. In addition, membranes were analyzed with rabbit monoclonal anti-human α2 (EPR 5788, Abcam), mouse monoclonal anti-human α1 antibody (MAB 5676, R&D Systems), mouse GAPDH antibody 6C5 (sc-32233, Santa cruz biotechnology) and mouse anti-β-actin IgG (AC-74, Sigma-Aldrich) to confirm the unchanged levels of integrin α1 or α2 protein. MRC5 protein lysates were used as positive controls.

### Image analysis

Focal adhesions were quantified as described previously^63^. Briefly, raw images were subjected to background correction with a rolling ball radius of 50 using ImageJ. Image contrast was enhanced using ImageJ plugin CLAHE and threshold adjusted. The number of focal adhesions, area of individual focal adhesion and total area of focal adhesions were quantified for a single cell. Cell spreading was quantified by measuring the cell surface area, which was in turn calculated by drawing the cell boundary using ImageJ. Spheroid images were also subjected to background correction and contrast enhancement using CLAHE plugin in ImageJ. Threshold adjusted images were used to quantify the radial cell density profile from the center of the spheroid, using the Radial Profile plugin from ImageJ. The cell densities of migrated cells at different distant points were used to quantify the distance of migration from the center of the spheroid as described earlier^64^.

### Statistical analysis

Statistical significance was assessed by using two tailed, unpaired t-tests as indicated in the figure legends and P < 0.05 considered statistically significant. Statistical analysis and all graphs were done using GraphPad Prism 5 software (GraphPad Inc, USA). Data normalization was done based on maximum value in each experiment and data from three independent experiments was pooled together, and average ± standard deviation was calculated for each cell type and condition. For cell adhesion assay, data normalization was done based on the average absorbance values of the fibronectin-coated wells in each experiment.

## Supplementary information

Supplementary data

## Data Availability

No datasets were generated or analyzed during the current study.
