# Supplementary material for: Integrin α11 cytoplasmic tail is required for FAK activation to initiate 3D cell invasion and ERK-mediated cell proliferation
Source: Sci Rep. 2019 Oct 25;9:15283. doi: 10.1038/s41598-019-51689-6 (PMC6814791; doi:10.1038/s41598-019-51689-6)
Supplement: Supplementary file 1 — Supplementary data [file 41598_2019_51689_MOESM1_ESM.pdf]

Supplementary information

Integrin α11 cytoplasmic tail is required for FAK activation to initiate 3D cell invasion and ERK-mediated cell proliferation

Pugazendhi Erusappan[1,2], Jahedul Alam[1], Ning Lu[1], Cédric Zeltz[1,3] and Donald Gullberg[1, *]

Cloning and plasmid construction

To generate a chimeric ITGA11-EGFP cDNA expression constructs in which EGFP was expressed in the C-terminal of the integrin α11 cytoplasmic tail via a 10 aminoacid linker sequence. The pBJ1-ITGA11 construct[1] was used as a template, and the stop codon of ITGA11 cDNA was replaced with a Kpn I restriction site. The ITGA11 cDNA without the stop codon was then subcloned into the BglII and Kpn I site of pEGFP-N3 vector, resulting in a cDNA fragment encoding the α11 with the EGFP-tag in the C-terminal. To improve expression levels, the ITGA11-EGFP cDNA was cloned back to pBJ1 vector (pBJ1-ITGA11-EGFP). The pBJ1- $_{Hu}$α11-1171-EGFP was generated using PCR-based site-directed mutagenesis by Mutagenex Inc and the constructs were confirmed by sequencing before use.

Generation of integrin α11-specific monoclonal antibodies

Mabs were produced using established procedures. NT-HRM mice (nanoTools Antikoerpertechnik, Germany) were immunized with human α11β1 integrin (R&D Systems), boosted twice and cell fusion was performed on day 68. Luminex beads coated with α11β1 integrin were used to screen α11 binders. Supernatants from FACS-positive clones (hybridoma supernatants reactive with mouse C2C12-human α11 cells, but not with cells lacking α11 (parental C2C12 cells), nor with cells lacking human α11 but expressing human β1 integrin; A431 cells)

were tested for their ability to immunostain α11 in western blotting. The mAb 210F4 was identified as one α11-specific hybridoma and has been selected for further characterization and used in this study (**Supplementary figure 1**).

References

1 Tiger, C. F., Fougerousse, F., Grundstrom, G., Velling, T. & Gullberg, D. alpha11beta1 integrin is a receptor for interstitial collagens involved in cell migration and collagen reorganization on mesenchymal nonmuscle cells. *Dev Biol* **237**, 116-129, doi:10.1006/dbio.2001.0363 (2001).

Supplementary figure legends

Supplementary figure 1. Characterization of α11 monoclonal antibody mAb 210F4. A-B. Wildtype C2C12 mouse myoblasts and C2C12 cells over-expressing human integrin α11 were stained with mAb 210F4 for 1 hour at 4°C. Cells were then washed and stained with goat anti-mouse R-Phycoerythrin IgG. Samples were analysed by flow cytometry using Intellicyt iQue and fluorescence intensity was measured. C. Western immunoblotting using the α11 mAb 210F4 on lysates from C2C12 cells expressing human integrin α2 ($_{Hu}\alpha 2$-C2C12) or human integrin α11-EGFP ($_{Hu}\alpha 11$-C2C12). β-actin is used as loading control.

Supplementary figure 2. Role of α11 tail in cell spreading. Cells were allowed to attach collagen I for 120 mins. Cells were fixed with 4% PFA and imaged using TIRF microscopy. Area of cell spreading was quantified. Results were expressed as mean ± standard deviation of at least three replicates from one representative experiment of at least three independent experiments. Statistical

significance was assessed by two tailed, unpaired t-tests and P-values are expressed as ***, $P<0.001$; **, $P<0.01$ and *, $P<0.05$.

Supplementary figure 3. Integrin α11 contributes to FAK and ERK activation

A. Human gingival fibroblasts (hGFs) were transfected with control (ctrl) siRNA or α11 siRNA (Individual) and 48 hours post transfection, cells were serum-starved and plated on collagen I in serum-free conditions. After 30 mins, cells were lysed, and the lysates were analyzed by western blotting. Protein bands were quantified by densitometry analysis. Statistical significance was assessed by two tailed, unpaired t-tests and P-values are expressed as ***, $P<0.001$; **, $P<0.01$ and *, $P<0.05$. **B.** Western blot showing protein levels of integrin α1 chain in hGFs treated with (Ctrl) siRNA or α11 siRNA (SMARTpool or Individual). **C.** Western blot showing protein levels of integrin α2 chain in hGFs treated with (Ctrl) siRNA or α11 siRNA (SMARTpool or Individual).

Supplementary figure 1

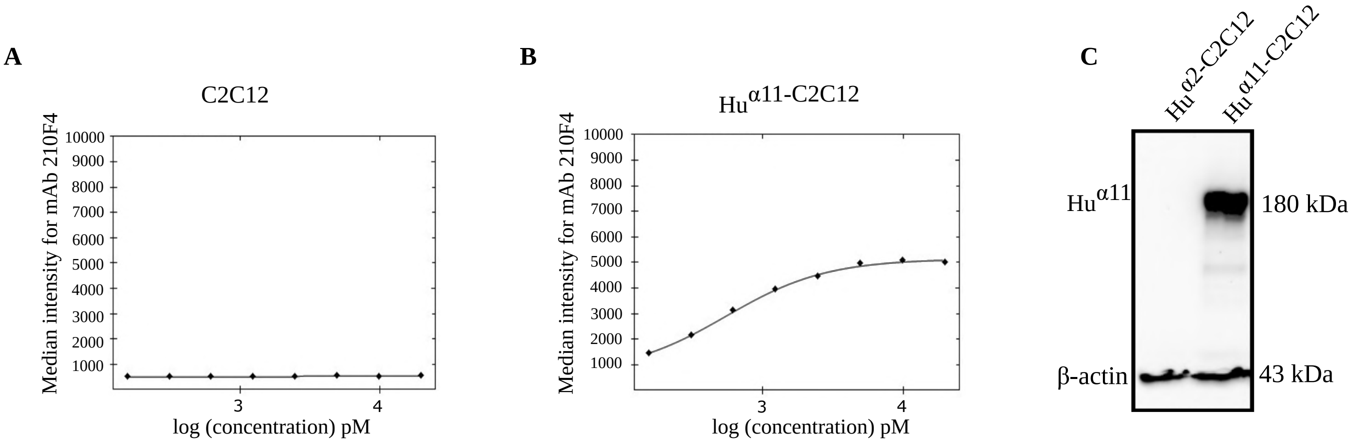

Supplementary figure 2

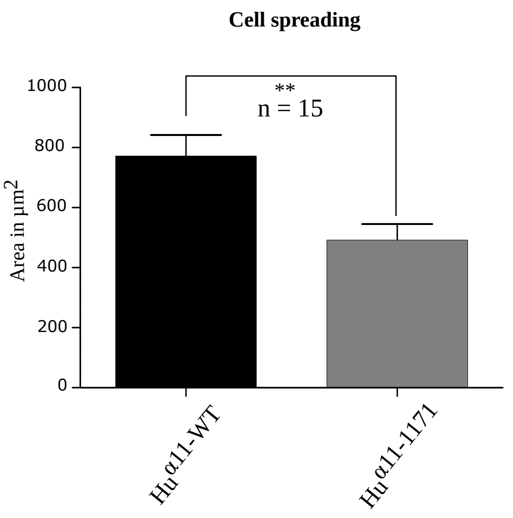

Supplementary figure 3

A

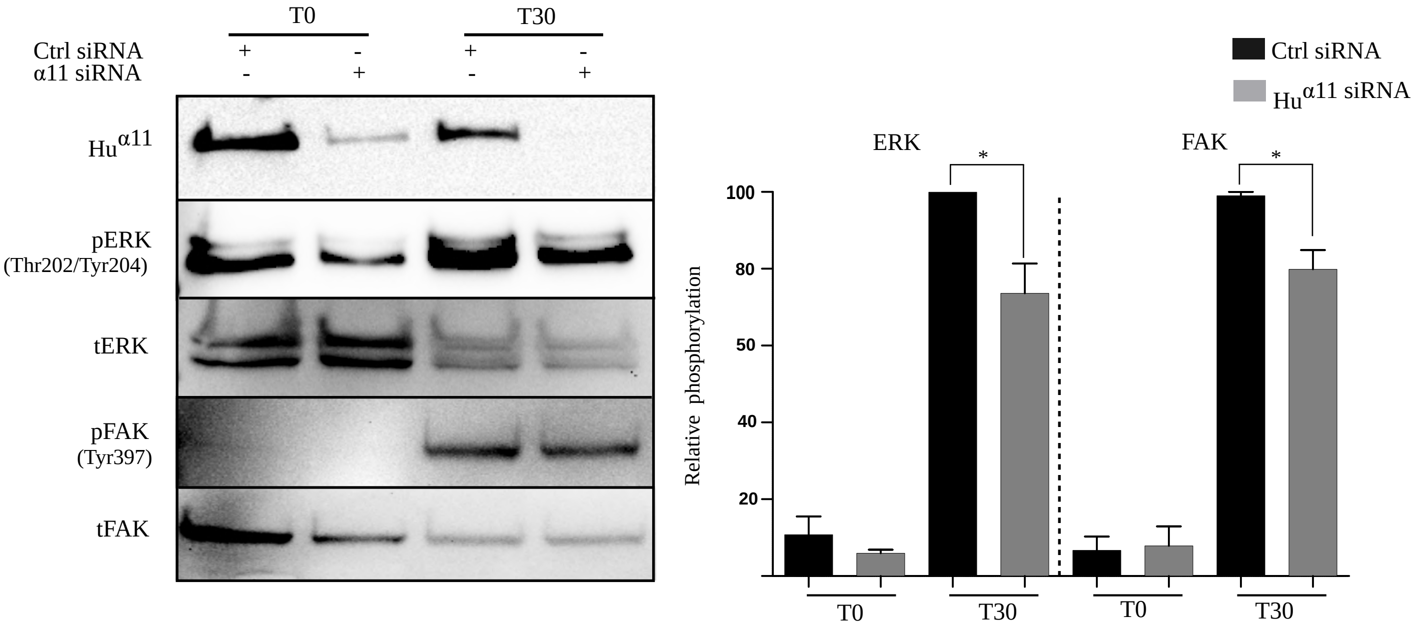

B

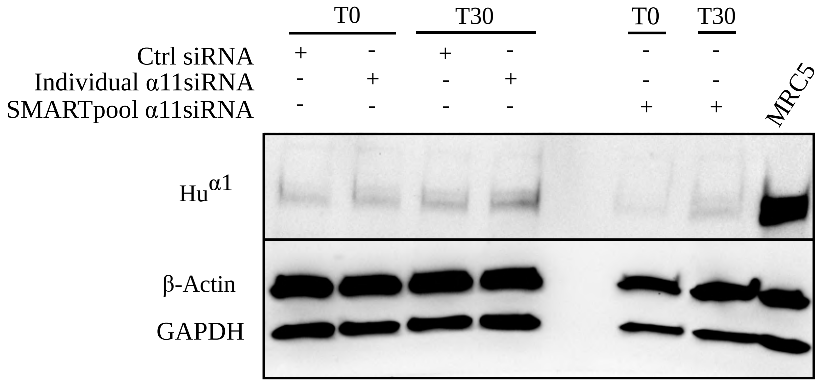

C

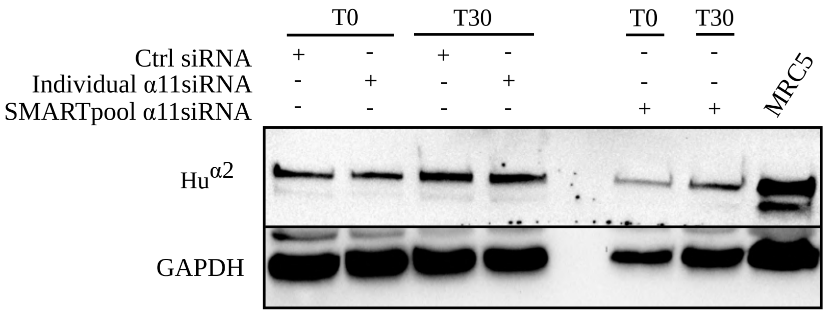

Full size western blots:

Figure 1B

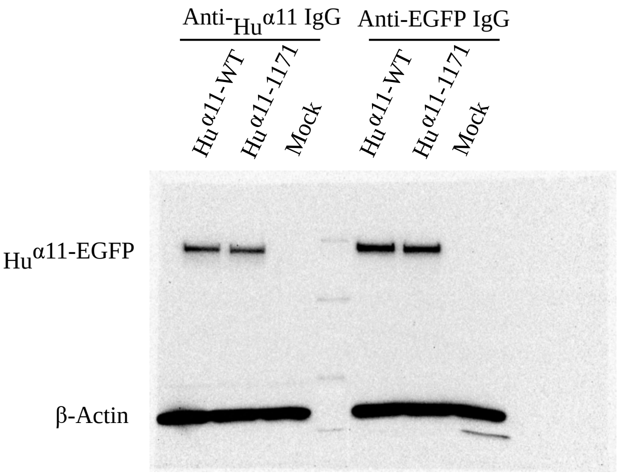

Figure 3A

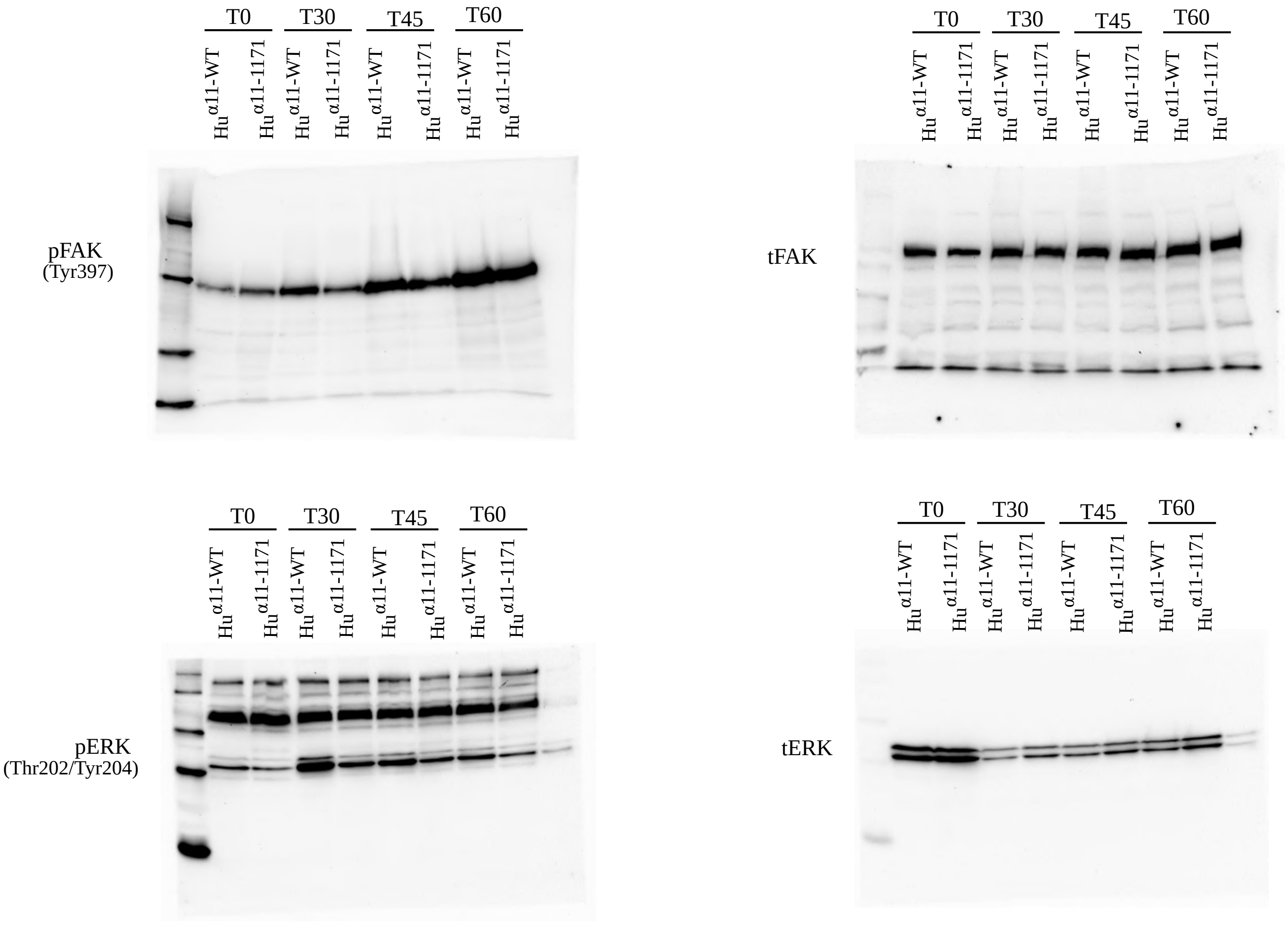

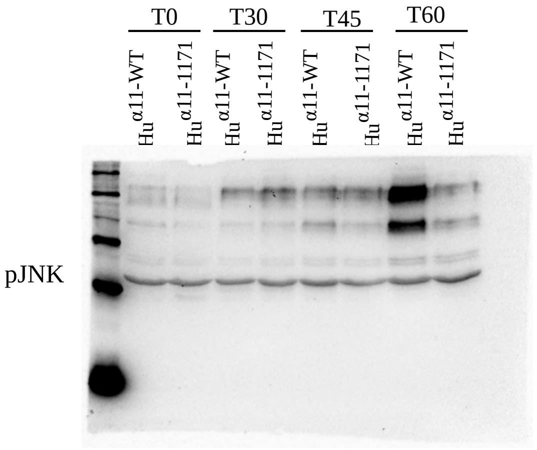
T0
T30
T45
T60
Huα11-WT
Huα11-1171
Huα11-WT
Huα11-1171
Huα11-WT
Huα11-1171
Huα11-WT
Huα11-1171
pJNK

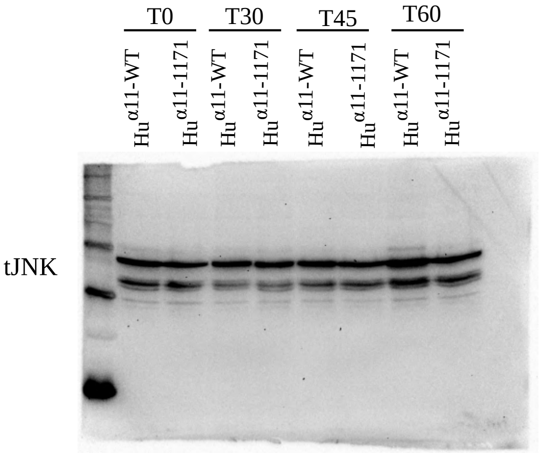
T0
T30
T45
T60
Huα11-WT
Huα11-1171
Huα11-WT
Huα11-1171
Huα11-WT
Huα11-1171
Huα11-WT
Huα11-1171
tJNK

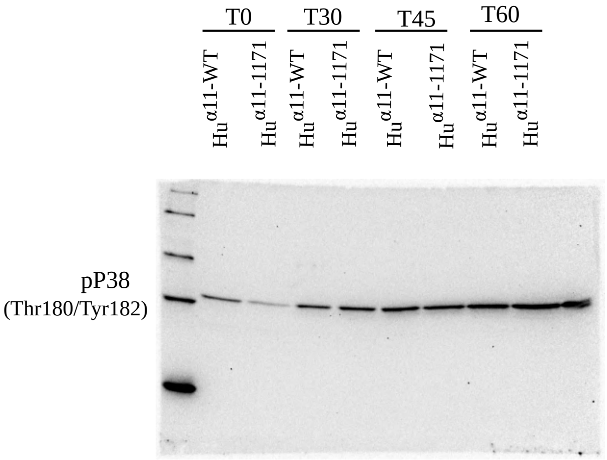
T0
T30
T45
T60
Huα11-WT
Huα11-1171
Huα11-WT
Huα11-1171
Huα11-WT
Huα11-1171
Huα11-WT
Huα11-1171
pP38
(Thr180/Tyr182)

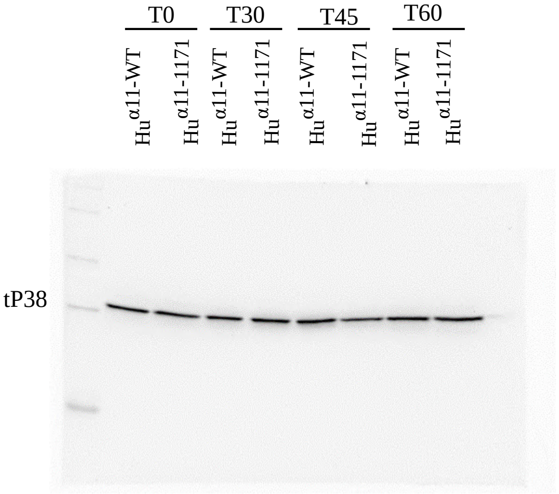
T0
T30
T45
T60
Huα11-WT
Huα11-1171
Huα11-WT
Huα11-1171
Huα11-WT
Huα11-1171
Huα11-WT
Huα11-1171
tP38

Figure 3B

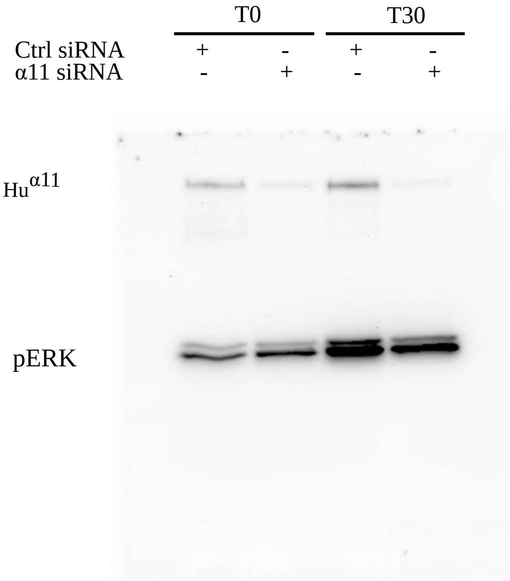
T0
T30
Ctrl siRNA + - + -
α11 siRNA - + - +
Huα11
pERK

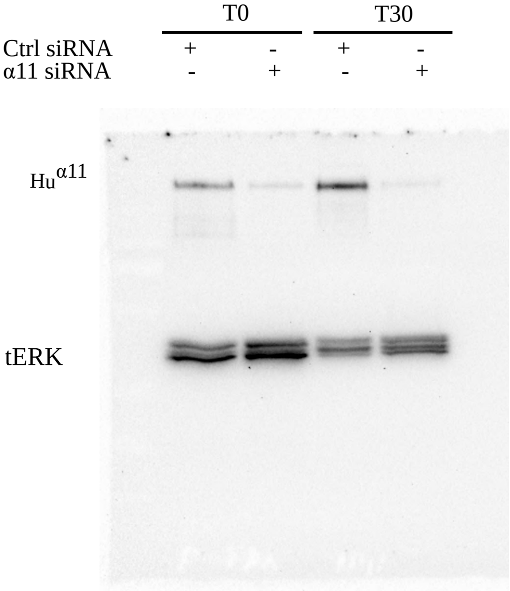
T0
T30
Ctrl siRNA + - + -
α11 siRNA - + - +
Huα11
tERK

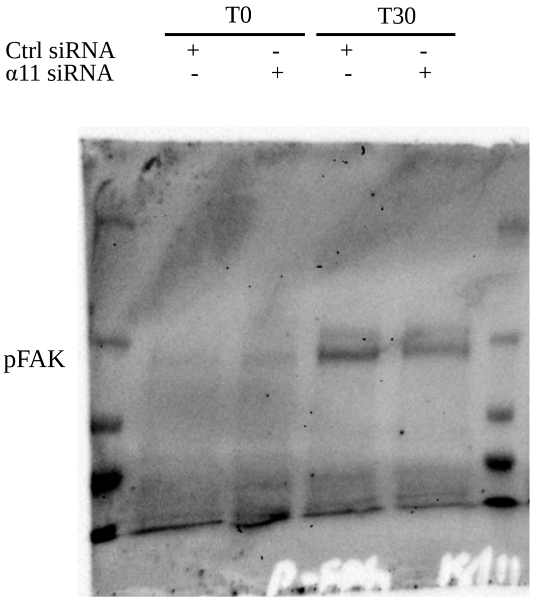
T0
T30
Ctrl siRNA + - + -
α11 siRNA - + - +
pFAK

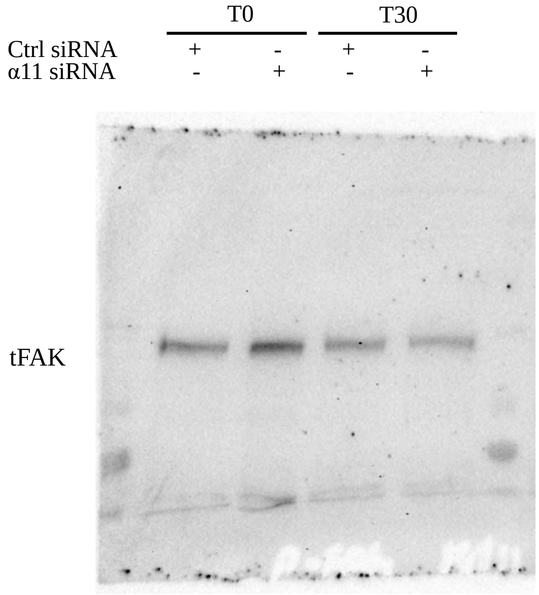
T0
T30
Ctrl siRNA + - + -
α11 siRNA - + - +
tFAK

Figure 4B

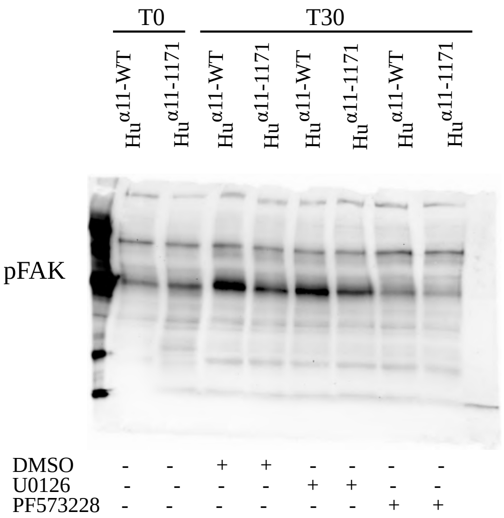
T0
T30
Huα11-WT
Huα11-1171
Huα11-WT
Huα11-1171
Huα11-WT
Huα11-1171
Huα11-WT
Huα11-1171
pFAK
DMSO - - + + - - - -
U0126 - - - - + + - -
PF573228 - - - - - - + +

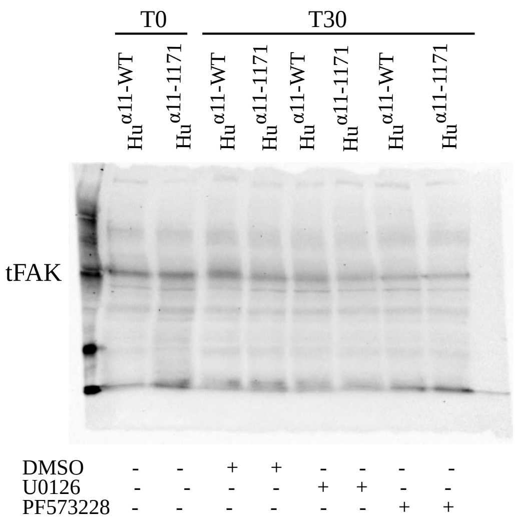
T0
T30
Huα11-WT
Huα11-1171
Huα11-WT
Huα11-1171
Huα11-WT
Huα11-1171
Huα11-WT
Huα11-1171
tFAK
DMSO - - + + - - - -
U0126 - - - - + + - -
PF573228 - - - - - - + +

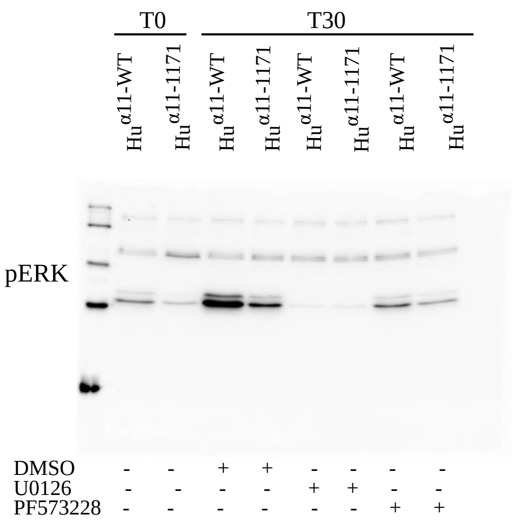
T0
T30
Huα11-WT
Huα11-1171
Huα11-WT
Huα11-1171
Huα11-WT
Huα11-1171
Huα11-WT
Huα11-1171
pERK
DMSO - - + + - - - -
U0126 - - - - + + - -
PF573228 - - - - - - + +

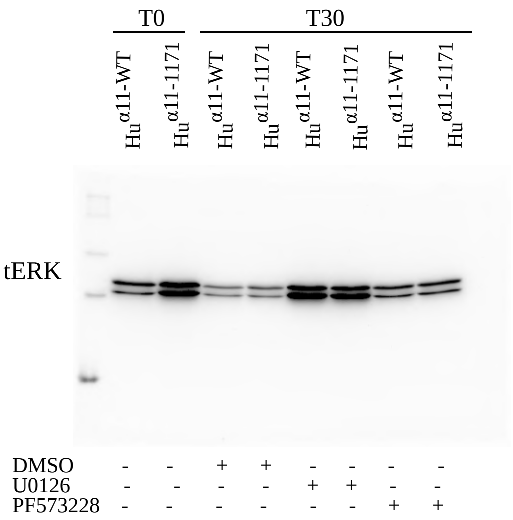
T0
T30
Huα11-WT
Huα11-1171
Huα11-WT
Huα11-1171
Huα11-WT
Huα11-1171
Huα11-WT
Huα11-1171
tERK
DMSO - - + + - - - -
U0126 - - - - + + - -
PF573228 - - - - - - + +

Supplementary figure 3

A

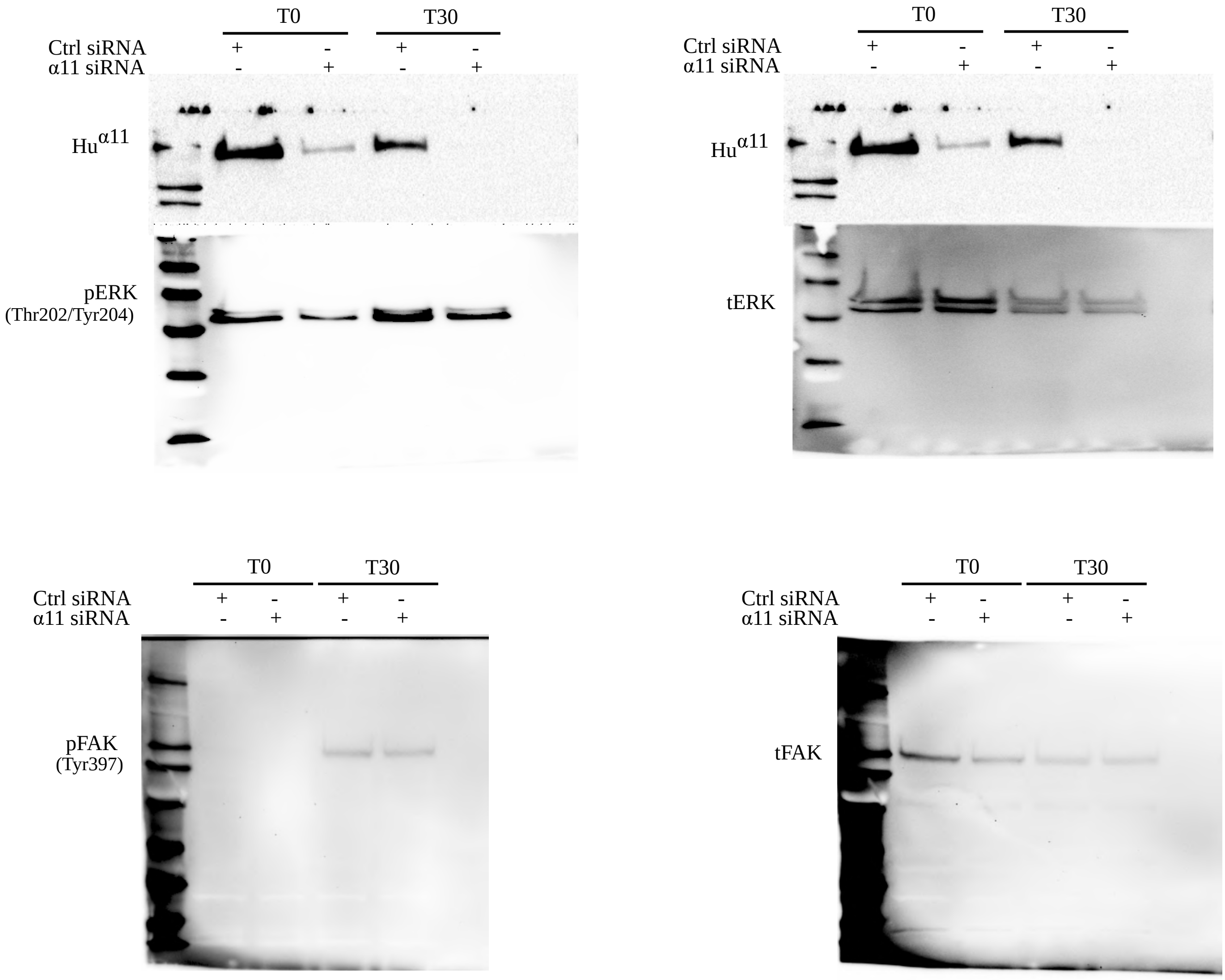

B

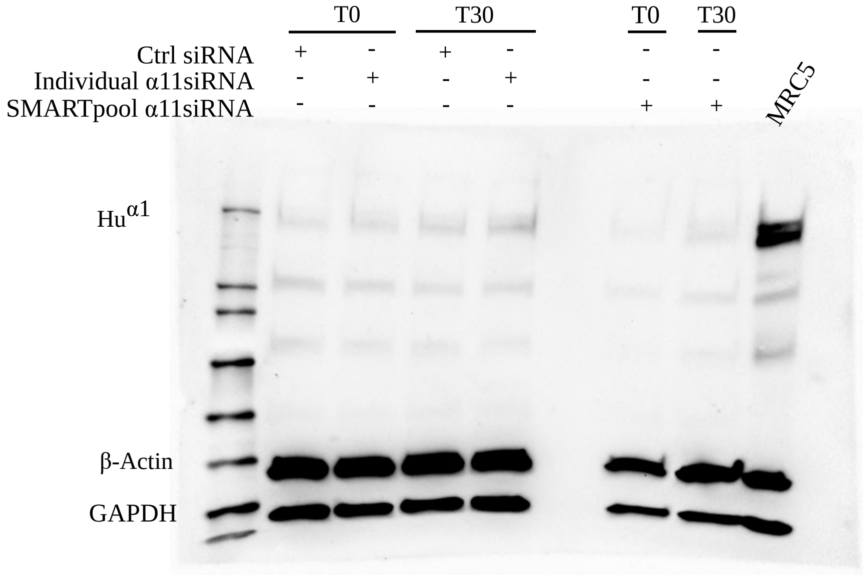

C

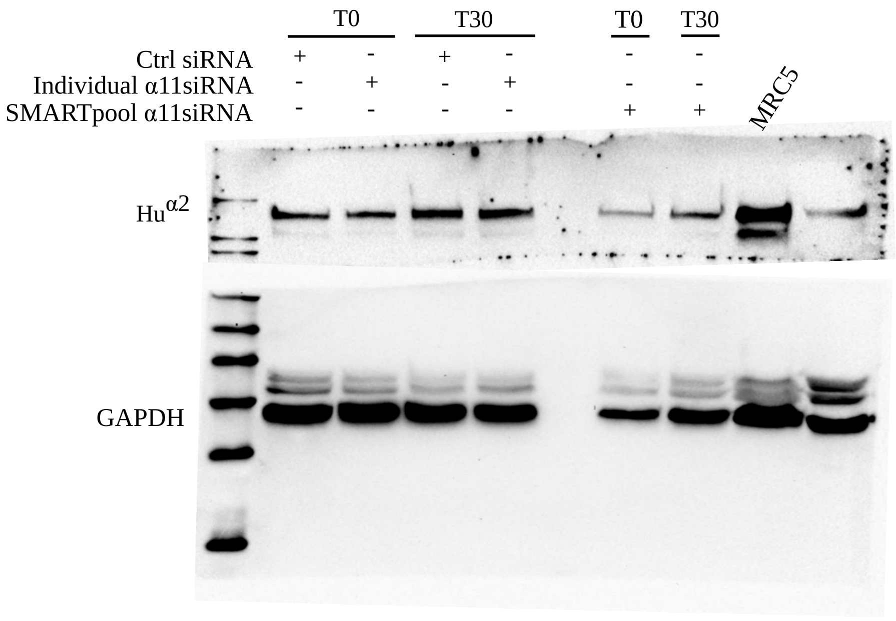
T0
T30
T0
T30
Ctrl siRNA + - + - - -
Individual α11siRNA - + - + - -
SMARTpool α11siRNA - - - - + +
MRC5
Hu$^{\alpha2}$
GAPDH
